# Non-technical factors on ophthalmology education: a narrative review

**DOI:** 10.3389/fmed.2024.1468631

**Published:** 2024-11-15

**Authors:** Yang Jiang, Hanyu Jiang, Zhikun Yang, Ying Li, Youxin Chen

**Affiliations:** ^1^Department of Ophthalmology, Peking Union Medical College Hospital, Chinese Academy of Medical Sciences, Beijing, China; ^2^Key Laboratory of Ocular Fundus Diseases, Chinese Academy of Medical Sciences and Peking Union Medical College, Beijing, China; ^3^Eight-Year Medical Doctor Program, Chinese Academy of Medical Sciences and Peking Union Medical College, Beijing, China

**Keywords:** ophthalmology education, non-technical factors, training, educators, trainees

## Abstract

Ophthalmology education is increasingly influenced by non-technical factors. This paper examines the multifaceted influences on ophthalmology education, focusing on direct and indirect factors that have shaped the training and wellbeing of ophthalmology students and residents. A systematic search of PubMed and Embase was carried out, searching date was from inception to 01/07/2024. A total of 8,232 articles were screened, of which 7,976 were excluded following abstract review. After reading the remaining 256 articles in full, a further 228 were excluded. A total of 28 original articles were included in this systematic review. The non-technical factors that influenced ophthalmology education included various crisis, inadequate curricular time, training resources, lack of training standardization and shortage of financial support and teaching resources. The review summarizes the influences on ophthalmology education of various non-technical factors, thereby helping educators improve the training methods.

## 1 Introduction

Ophthalmology education, traditionally centered around technical skills and clinical expertise, is increasingly influenced by a myriad of non-technical factors. These factors, encompassing mental health, economic conditions, gender dynamics, intrinsic motivation, and communication skills, play a crucial role in shaping the educational landscape for future ophthalmologists. Understanding these influences is essential for developing comprehensive training programs that address not only the technical competencies but also the holistic development of medical professionals.

Recent global events, such as the COVID-19 pandemic, have underscored the importance of considering non-technical influences in medical education. The pandemic has brought to light the significant psychological toll on students and residents, disrupting traditional educational pathways and necessitating a shift to virtual learning environments. Concurrently, economic crises and other societal disruptions have further strained educational resources and opportunities, highlighting the need for resilient and adaptable educational frameworks.

Gender dynamics within ophthalmology education also warrant attention. Studies indicate that gender-related factors influence career choices, mentorship opportunities, and educational experiences, thereby impacting the overall diversity and inclusivity within the field. Additionally, intrinsic motivation and self-determination are pivotal in fostering effective learning environments. The creation of supportive educational settings that meet students’ psychological needs can enhance their engagement and professional growth.

Effective communication skills are equally vital in medical training, influencing patient interactions and clinical outcomes. As such, the development of these skills should be integral to ophthalmology education, ensuring that graduates are not only technically proficient but also adept at building rapport with patients.

This research aims to explore the diverse non-technical factors impacting ophthalmology education, drawing on recent studies and surveys to provide a comprehensive understanding of their implications. By examining these influences, we seek to inform the development of more holistic and adaptive educational strategies that can better prepare future ophthalmologists for the complexities of their profession.

## 2 Materials and methods

### 2.1 Eligibility criteria

All original studies were evaluated if they described development in ophthalmic training. Studies were included: (1) study participants were ophthalmologists or medical students who were accepting their ophthalmology training; (2) studies related to non-technical factors. Studies were excluded: (1) if they did not provide original data; (2) articles not specific to ophthalmology; (3) studies not related to training or education. We included papers written in English.

### 2.2 Search methods

A systematic search of PubMed and Embase was carried out, using the terms “(train* OR education OR learning) AND ophthalm*”. Search date was from inception to 01/07/2024. Reference lists from included articles and relevant reviews were hand searched for eligible studies.

### 2.3 Study selection

**FIGURE 1:**
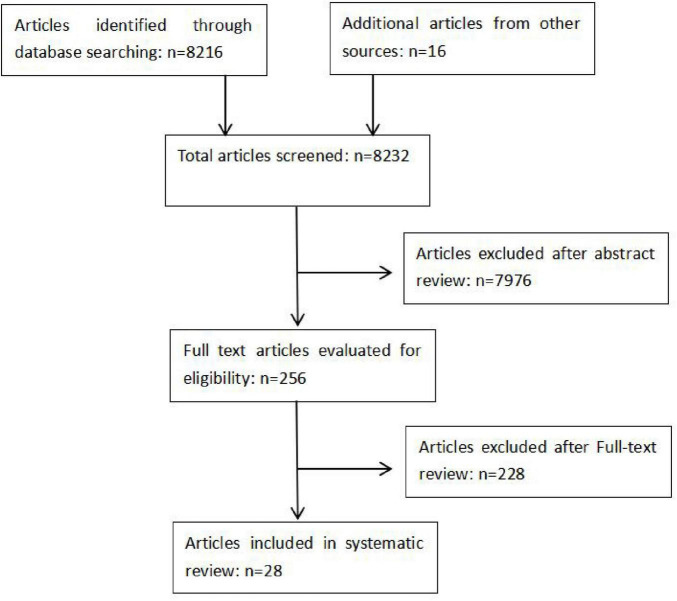
Flow diagram of study selection process.

Two authors, YJ and HJ, carried out independent, duplicate searches. All abstracts were reviewed and articles that were potentially eligible were read in full. A final list of studies meeting the eligibility criteria was compared and disagreements resolved by discussion (Figure 1).

### 2.4 Data collection

The same two authors extracted data for each study separately and differences were resolved through discussion. Data collected included details of the observation subject, sample size, methods and evaluation results.

## 3 Results

A total of 8,232 articles were screened, of which 7,976 were excluded following abstract review. After reading the remaining 256 articles in full, a further 228 were excluded. A total of 28 original articles were included in this systematic review (Figure 1). Details of findings are summarized in Tables 1–4 according to the different non-technical factors on ophthalmology education.

**TABLE 1:** Direct factors of non-technical factors on ophthalmology education.

References	Country/ Region	Observation subject	Sample size	Methods	Evaluation results
Pang et al. (1)	The United States and Canada	Eye care professionals and students	812 participants	Depression, anxiety, and psychological stress were assessed using validated questionnaires	Students had less reduction of stress than other eye care professionals (all *P* < 0.05)
Aragão et al. (2)	Brazil	Residents and fellows in ophthalmology training	271 participants	A 42-questions survey elaborated by the authors	Higher stress scores were identified in residents and fellows that had their surgical training interrupted during the pandemic (*P* < 0.001)
Mahalingam et al. (3)	India	Ophthalmology residents and fellows	265 participants	Depression, Anxiety, and Stress Scale—21 items (DASS-21) and Brief Coping Orientation to Problems Experienced (COPE)	50.2% (*n* = 133) had anxiety and 36.6% (*n* = 97) had stress
Ní Gabhann-Dromgoole et al. (4)	Irish	4th year senior cycle medical students enrolled on an ophthalmology clinical attachment	243 participants	A validated course evaluation questionnaire (CEQ) and exam score	Compared to traditional delivery, students were dissatisfied with the level of choice afforded by the online flipped classroom. However, no significant difference in exam score was observed between two groups.
Balci et al. (5)	Türkiye	Ophthalmology residents	458 participants	A web-based survey consisting of 28 questions	There were significant decreases in the number of patients examined by resident doctors and theoretical training time compared to Pre Pandemic (*p* < 0.05). In the examinations held in the clinic, in the 1st year of the pandemic, the grade average was lower than before the pandemic (*p* < 0.05).
Chen et al. (8)	China	Undergraduate ophthalmic students	46 participants	This study compared students’ perspectives and study outcomes of the Self-directed learning (SDL) and traditional classroom learning (TCL) to preliminarily investigate the effect of SDL in undergraduate ophthalmology education	There was no difference in student’s satisfaction with the two learning models (*p* = 0.83). There was no statistical difference in post-class tests scores (71.37 ± 3.59 vs. 72.96 ± 2.91, *p* > 0.05)
Mishra et al. (9)	India	Trainee ophthalmologists	716 participants	An online survey	A large majority 578/716 (80.7%) of the trainees agreed that the lockdown had a negative impact on their surgical training; 62.4% of the residents felt there was 50% or more reduction in their surgical training during the lockdown
Ghannam et al. (10)	Lebanon	Ophthalmology residents	52 participants	An online questionnaire	70% of the residents reported that their clinical and operative training has been affected. The economic crisis was considered the major factor impacting the training programme by 74% of the residents

**TABLE 2:** Indirect factors of non-technical factors on ophthalmology education.

References	Country/ region	Observation subject	Sample size	Methods	Evaluation results
Aljuhani et al. (11)	Saudi Arabia	Medical students and interns	449 participants	A self-administered questionnaire	The leading factor to be fewer working hours for the males and potential for a good work-life balance for the females, whatsoever there was no significant difference between males and females when choosing ophthalmology as a future specialty
Paul et al. (13)	the United States	Association of University Professors of Ophthalmology (AUPO) chairs, program directors (PDs), and medical student educators (MSEs)	75 participants	A 22-question survey	21.2% of male versus 56.1% of female members agreed that a mentee of the same gender was important (*P* < .01); Females were more likely to feel gender-specific mentorship was important
Dutt et al. (14)	-	Penultimate year medical students	10 participants	Interview questions were developed using the seven-step process outlined in the literature	Certain factors contributed heavily to a certain conception of one or more particular psychological need, 81% of factors were influenced by students’ learning environment
Li et al. (15)	China	Ophthalmology training residents	261 participants	Questionnaires	The demeanor of training residents toward patients (*p* < 0.001), and the quality of doctor–patient communication (*p* < 0.001) significantly varied between the groups whether the patients are willing to undergo an indirect ophthalmoscopy examination by resident doctors, or not

**TABLE 3:** Non-technical factors in ophthalmic training.

References	Training program/ training device	Observation subject	Design	Assessment methods	Results
Feudner et al. (16)	Capsulorhexis/the Eyesi (VRmagic, Mannheim, Germany) ophthalmic virtual reality surgical simulator	31 medical students and 32 ophthalmological residents in postgraduate year (PGY) 1 to 5	Randomized, masked experimental study	Each capsulorhexis was evaluated with regard to five criteria (circularity, size, centering, time, tissue protection) using a predefined scoring system with a maximum overall score of 10 points (2 per criterion)	Residents also showed marked improvement for time scores whereas no obvious difference in time scores was observed for students; for students and residents taken together, subjects with a low number of attempts on training had a higher probability to perform well than the group with a higher number of attempts
Cissé et al. (17)	Vitreoretinal modules/the Eyesi (VRmagic, Mannheim, Germany) ophthalmic virtual reality surgical simulator	Fifteen residents with no vitreoretinal experience and six trained vitreoretinal surgeons (> 100 procedures per year)	A prospective controlled interventional study	The simulator software calculates a performance score for each level, based on five criteria: target achievement, efficiency, instrument handling, microscope handling and tissue treatment; a maximum total of 100 points can be achieved in each task and points are deducted for each error that occurs	Experienced vitreoretinal surgeons outperformed residents with regard to the overall score on the navigation 1 (*p* = 0.01), forceps 1 (*p* < 0.01), epiretinal membrane peeling modules 1 and 2 (*p* = 0.02) and ERM2 (*p* = 0.04) modules; experienced surgeons slightly improved or achieved similar scores in all modules; in contrast, novices obtained similar or higher scores in only four modules
Jaud et al. (18)	Vitreoretinal surgical skills/the Eyesi (VRmagic, Mannheim, Germany) ophthalmic virtual reality surgical simulator.	10 junior residents without any surgical experience, 8 senior residents with prior experience in cataract surgery and 5 vitreoretinal surgeons	A prospective controlled interventional study	The simulator software calculates a performance score for each level, based on five criteria: target achievement, efficiency, instrument handling, microscope handling and tissue treatment	Senior residents significantly improved their simulator skills over time, reaching a plateau at the fifth iteration and equalling expert performance (*p* = 0.420)
Mondal et al. (19)	Vitreoretinal surgical training/the Eyesi (VRmagic, Mannheim, Germany) ophthalmic virtual reality surgical simulator.	Retina fellows-in-training	A multicenter survey	A questionnaire consisting of 22 questions	Most (*n* = 25, 68%) respondents considered surgical simulators to be the best training tool before operating on the human eye; the majority (*n* = 33, 89%) of participants responded that VR surgical skills acquired during simulator training were transferrable to the operating room
Staropoli et al. (20)	Learning cataract surgery/the Eyesi (VRmagic, Mannheim, Germany) ophthalmic virtual reality surgical simulator	The second year of ophthalmology residency for novice postgraduate year 3 (PGY-3) residents	A retrospective consecutive case series	Complication rates: posterior capsule tear (PCT) rate and vitreous prolapse rate; a questionnaire about residents’ experience of the surgical simulator	The addition of surgical simulation training was associated with a significantly reduced rate of complications, including PCTs and vitreous prolapse, among novice PGY-3 residents; in regards to how closely the modules resembled live surgery (scale of 1–5, with 5 = “extremely well”) the residents rated the capsulorhexis module 3.45, sculpt experience 3.18, and quadrant removal 3.1
Colin et al. (21)	Continuous, curvilinear capsulorhexes (CCCs) during cataract surgery/the Eyesi (VRmagic, Mannheim, Germany) ophthalmic virtual reality surgical simulator	Resident surgeons at a teaching hospital with level of postgraduate year (PGY)	Retrospective educational interventional case series	The rates of errant capsulorhexes were compared	There was a statistical trend toward fewer errant CCCs among PGY 4 (14.6%) compared with PGY 3 (22.8%) surgeons (*P* = 0.12)
Le et al. (22)	A practice trial in the anterior segment training module, followed by 3 scored trials in the anterior forceps, antitremor, and capsulorhexis modules/the Eyesi (VRmagic, Mannheim, Germany) ophthalmic virtual reality surgical simulator	4 medical students, 4 ophthalmic medical technologist trainees, 36 ophthalmology residents, 3 fellows, and 18 staff ophthalmologists	Multicentre cross-sectional study	Each task was scored out of 100, and points were deducted or attributed according to the criteria set out by the simulator computer software	Participants with greater experience achieved significantly higher total scores than those who were less experienced (*p* = 0.011), with lower total task time (*p* = 0.044) and fewer injuries to the cornea (*p* = 0.001) and lens (*p* = 0.026)
Sikder et al. (23)	Capsulorhexis Training of cataract surgery/MicroVis Touch (ImmersiveTouch, Chicago, IL, USA)	78 ophthalmology residents	A prospective study	Four variables (circularity, accuracy, fluency, and overall) were tested by the simulator and graded on a 0–100 scale	The improvement in all test variables was statistically significant (*P* < 0.05)
Lam et al. (24)	Four main phacoemulsification cataract surgery procedures–(1) corneal incision (CI), (2) capsulorhexis (C), (3) phacoemulsification (P), and (4) intraocular lens implantation (IOL)/prototype	10 experienced ophthalmologists and 6 medical residents	A prospective study	A set of parameters, which monitor the training objectives in each procedure of cataract surgery, were selected and implemented into the performance evaluation system of the simulator to assess the performance of the participants	Subjects with greater experience obtained significantly higher scores in all four main procedures; Positive correlation was observed between experience and anti-rupture
Weiss et al. (25)	Endoscopic endonasal dacryocystorhinos tomy training/the endoscopic endonasal surgery simulator	Ophthalmology residents	A prospective study	Performance on these tasks was videotaped and graded by 2 masked observers	Residents who trained on the simulator performed significantly better compared with the group who trained on cadaver
Famery et al. (26)	Descemet membrane endothelial keratoplasty (DMEK)/Artificial chamber and 3D printed iris	3 ophthalmology residents with no DMEK experience (group B) and 2 cornea fellows (group A)	A prospective study	A complete DMEK surgery gives 100 points of which 10 points were subtracted if incisions were not located correctly, flush or massage were needed, if bumping or air injection for graft manipulation were performed, if the graft was decentred or upside down or if there was an injured epithelium	Performance score ranged from 90 to 100 in group A and from 22 to 75 in group B with a respective mean of 94 and 52; difference between the two groups was found to be statistically significant *p* = 0.03

**TABLE 4:** Problems and shortcomings on ophthalmology education.

References	Country/ region	Observation subject	Sample size	Methods	Evaluation results
Gostimir et al. (28)	Canada	Undergraduate program directors	7 participants	An online survey	Only 5 of 14 (35.7%) schools were found to have a mandatory clinical clerkship ophthalmology rotation; In each case, the mandatory rotation is less than 2 weeks
Moxon et al. (29)	The United States	Individuals responsible for medical student education, as indicated in the Association of University Professors of Ophthalmology (AUPO) directory	95 participants	A survey via e-mail and telephone	In terms of clinical exposure, only 16% of schools required clinical rotations, half of which were embedded within another course
Adewara et al. (30)	Nigeria	Ophthalmology trainers	158 participants	A nationwide web-based survey	51.9% of respondents rated the volume of surgery as “less or much less” than adequate (*n* = 82) and 22.8% of respondents rated the impact of emigration of ophthalmologists on training as “very negative” (*n* = 36)
Scott et al. (31)	Australia	The relevant individual per institution responsible for curriculum	19 participants	A national survey encompassed 35 questions on student demographics, teaching methods, core theoretical topics, clinical skills, and assessment methods in ophthalmology	Ophthalmology rotations were required in 63.3% (*n* = 12), while 36.7% (*n* = 7) did not have mandatory terms; all respondents reported student exposure to at least one clinical day in ophthalmology, with total teaching time ranging from less than six hours (36.9%), up to greater than two weeks (10.5%)
Tsai et al. (33)	The Asia-Pacific (APAC), including 2 regions, namely Western Pacific (WP) and Southeast Asia (SEA)	Young Ophthalmologist leaders from the national member societies of the Asia-Pacific Academy of Ophthalmology (APAO)	130 participants	An anonymous online survey	Among respondents in SEA, 14 (17.3%) did not have an official curriculum, while 5 (6.2%) did not know if they had one; among respondents in WP, 9 (18.4%) did not have one, while 1 (2%) did not know if they had one; most (98/130, 75%) indicated an interest for a common training standard across the APAC

### 3.1 Direct factors

#### 3.1.1 Coronavirus disease 2019

Pang et al. (1) conducted a longitudinal study to evaluate the mental health impact of the COVID-19 pandemic on ophthalmic personnel and students. The study identified students experiencing high stress levels. Aragão et al. (2) evaluated the impact of the COVID-19 pandemic on the mental health of ophthalmology residents and fellows in Brazil. Using a 42-question survey that included the Perceived Stress Scale (PSS-10), the study found that both residents and fellows experienced similar stress levels during the pandemic, but those who had their surgical training interrupted reported significantly higher stress levels. Mahalingam et al. (3) conducted a survey to evaluate the long-term impact of the COVID-19 pandemic on the training and mental health of ophthalmology residents and fellows in India. The study, which involved 265 participants, found a significant reduction in clinical exposure and surgical cases, with participants handling only 29.6–46.6% of the pre-pandemic volume. The study revealed high levels of depression, anxiety, and stress among participants, with 55.8% experiencing depression and 50.2% reporting anxiety. It has been demonstrated that COVID-19 pandemic had negative impact on mental health of ophthalmology residents.

Ní Gabhann-Dromgoole et al. (4) evaluated the shift to online learning for ophthalmology education during the COVID-19 pandemic. The study compared traditional delivery (TD) and online flipped classroom (OFC) approaches among 243 fourth-year medical students. Results indicated that OFC students reported reduced satisfaction with staff motivation, feedback, and clarity of work standards. They also found the OFC less beneficial for developing problem-solving skills. Balci et al. (5) examined the impact of the COVID-19 pandemic on ophthalmology residency education in Turkey. The study surveyed 37 educators across 35 centers, highlighting significant reductions in patient examinations, theoretical training, and surgical procedures during the pandemic compared to the pre-pandemic period. Most residents (53.71%) worked on COVID-19 duties, averaging 69.57 days away from their own clinics. Theoretical training shifted largely online, with many hospitals implementing physical distancing measures for in-person sessions. The study noted declines in academic publications and congress participation, alongside increased stress and resignations among residents. These findings underscore the pandemic’s severe disruptions to ophthalmology education and training. It has been demonstrated that COVID-19 pandemic had influenced the conventional ophthalmology education.

Martins et al. (6) addressed the challenges of teaching ophthalmology during the COVID-19 pandemic by developing a new methodology for teaching direct ophthalmoscopy and red reflex testing. The approach involved online theoretical classes using platforms like YouTube to provide tutorials and enable students to construct teaching dummies, thereby enhancing their understanding of the examination’s fundamental principles. Practical sessions used these dummies and portable handheld fundus cameras, allowing real-time supervision and reducing in-person class time without compromising teaching quality.

Patel et al. (7) explored the adaptations in medical education and ophthalmology residency interviews due to the COVID-19 pandemic. The study highlighted the shift to virtual platforms for residency interviews and educational activities. Social media usage by ophthalmology programs increased significantly, with Instagram and Twitter being the most popular. The study found that virtual tools like open houses and simulated internships helped maintain educational standards. Chen et al. (8) explored the efficacy of self-directed learning (SDL) as an alternative to traditional classroom learning (TCL) in undergraduate ophthalmic education during the COVID-19 pandemic in China. The findings revealed no significant difference in learning outcomes or student satisfaction between the two models. However, students with a high interest in ophthalmology found SDL more effective in enhancing self-study ability and learning efficiency, whereas TCL was preferred for developing problem-solving skills. Mishra et al. (9) reported that a large majority 578/716 (80.7%) of the trainees agreed that the lockdown had a negative impact on their surgical training, 62.4% of the residents felt there was 50% or more reduction in their surgical training during the lockdown. It has been demonstrated that COVID-19 pandemic had reduce the training opportunity for ophthalmology trainees.

#### 3.1.2 Economic crisis

Ghannam et al. (10) investigated the effects of the economic crisis, and the Beirut explosion on ophthalmology training in Lebanon. This observational cohort survey included 52 participants, revealing that the majority experienced significant disruptions in training and mental health challenges, including increased stress, anxiety, and depression. The study highlighted financial burdens, reduced patient loads, and a decrease in surgical and educational activities as primary issues.

### 3.2 Indirect factors

#### 3.2.1 Gender

Aljuhani et al. (11) authored the research, published in *Cureus* in 2023. This cross-sectional study investigates the influence of gender on the decision to pursue ophthalmology as a career among medical students and interns in Madinah, Saudi Arabia. Data from 449 participants revealed that while fewer working hours attracted males to ophthalmology, females were more interested in the anatomy and physiology of the eye. Huh et al. (12) authored the study published in *JAMA Ophthalmology* in 2023. The retrospective cross-sectional study examines gender differences in ACGME Milestone ratings among ophthalmology residents. Analyzing data from 452 residents, the study found no significant gender differences in patient care ratings at midyear or year-end. However, female residents had lower midyear medical knowledge ratings compared to male residents. The findings suggest the need for ongoing examination of potential biases in resident evaluations to ensure fair educational and career opportunities. Paul et al. (13) authored the research, published in the *American Journal of Ophthalmology* in 2022. The study evaluates attitudes toward mentorship of female medical students among ophthalmology educators. Using a cross-sectional survey, the research found that while both male and female respondents mentored a similar percentage of female students, female respondents placed higher importance on gender-specific mentorship. The study suggests that expanding female mentorship in ophthalmology could promote equity in training and address the lack of female representation in leadership roles.

#### 3.2.2 Intrinsic motivation

Dutt et al. (14) examined the factors influencing medical students’ autonomy, competence, and relatedness in ophthalmology education through the lens of Self-Determination Theory (SDT). The study, conducted at the University of Western Australia, identified that intrinsic motivation is crucial for effective learning and student contentment. The researchers highlighted the importance of creating environments that satisfy students’ basic psychological needs to foster intrinsic motivation, which in turn enhances learning outcomes and promotes continuous professional development.

#### 3.2.3 Communication skills

Li et al. (15) examined the willingness of outpatients in China to undergo fundus examinations conducted by ophthalmology residents as part of the National Standardized Training for Resident Doctors (STRD). The study, involving 261 valid responses, identified that patient cooperation was influenced by the demeanor of the residents, and the quality of doctor-patient communication. These findings suggest that improving communication skills among residents could significantly increase patient cooperation, thereby improving the effectiveness of ophthalmology training programs in China.

### 3.3 Non-technical factors in advanced ophthalmic training

In recent years, a large number of new techniques have emerged in ophthalmology education, including: virtual reality and physical models (e.g., three-dimensional 3D technology). Novel techniques can drastically improve the trainees’ clinical competence (16–26). However, among the novel training techniques, there were still non-technical factors that could affect ophthalmology education.

#### 3.3.1 Fatigued training could reduce learning effectiveness

Feudner et al. (16) reported that for students and residents taken together, subjects with a low number of attempts on training had a higher probability to perform well than the group with a higher number of attempts. Cissé et al. (17) reported that fatigue and loss of concentration over time may have affected residents’ performance, as demonstrated by the lower scores of the last tasks. They suggests that several short sessions are probably more effective than a single longer one.

#### 3.3.2 The high expense of the advanced training equipment posed a great barrier for their application in ophthalmology education

Despite evidence that novel technologies like virtual reality simulator-based surgical training yields positive outcomes, not all fellowship programs can afford to include simulator training in their curriculum owing to the high expense of the equipment and the need for periodic upgradation (19). According to La Cour et al. (27), this poses a great barrier to establishing a simulated training curriculum.

#### 3.3.3 Other non-technical limits

Mondal et al. (19) reported that the main reasons limiting the utilization of the simulators to their full capacity were the unavailability of the simulator beyond their working hours (54.8%), the lack of a structured training program (19.3%), and the scarcity of a dedicated supervisor (16.1%).

To sum up, the influence of various non-technical factors on the new technology of ophthalmology teaching clearly exists. Combining the non-technical factors discussed above, it is suggested that short sessions of training is recommended to the trainees, sufficient financial support and strong teaching resources are necessary to provide efficient ophthalmic training for the trainees.

### 3.4 Problems and shortcomings

#### 3.4.1 Lack of curricular and clinical rotation time

Gostimir et al. (28) assessed the state of undergraduate ophthalmology education in Canadian medical schools through a survey of program directors. The study found that only 35.7% of schools required a mandatory clinical rotation in ophthalmology, typically lasting less than two weeks. Moxon et al. (29) analyze the state of ophthalmology education for medical students in the United States. Their survey of 117 institutions reveals a decline in curricular time dedicated to ophthalmology, now often confined to preclinical years with supplemental extracurricular activities. The study highlights the importance of continuous adaptation in medical education to maintain ophthalmic training quality.

#### 3.4.2 Lack of training resources

Adewara et al. (30) conducted a web-based survey of ophthalmology trainers in Nigeria to assess the ophthalmology residency training. With a response rate of 71.2%, the survey highlighted that while the overall quality of training was rated as good, significant inadequacies were noted in surgical volume and resources. Trainers recommended improvements in funding, training resources, and examination practices.

#### 3.4.3 Lack of training standardization

Scott et al. (31) conducted a national survey on ophthalmology education in Australian medical schools. The survey, which included responses from 19 of 21 medical schools, found that 63.3% required ophthalmology rotations, while 36.7% did not. Chinese ophthalmology residency training is continuously evolving with an emphasis on standardization. Wang et al. (32) assesses the current status of ophthalmology residency training in China compared with that in the United States. It reveals significant regional differences among training programs due to the lack of a national standard, resulting in varying competencies of graduating Chinese ophthalmology residents. A survey of ophthalmology training experiences (33) among young ophthalmologists in the Asia-Pacific region highlights the diversity in training experiences across different countries. The study compares experiences between trainees from Southeast Asia and Western Pacific countries, revealing differences in training quality, exposure to various subspecialties, and satisfaction levels. The findings emphasize the need for standardized training curricula to ensure consistent competency among ophthalmologists in the region.

#### 3.4.4 The decline of ophthalmology education

Liao et al. (34) explores the current challenges and potential solutions in ophthalmology education within US and Canadian medical schools. Utilizing a systematic review of 66 articles, the study identifies four main themes: challenges in curriculum standardization, inadequate clinical exposure, the role of technology, and innovative teaching methods. Spencer et al. (35) conducted a systematic review to analyze trends in ophthalmology education in medical schools worldwide. This study reviewed 52 publications from 19 countries, highlighting a significant decline in course lengths over the past two decades, with averages dropping from 92.9 h in the 2000s to 52.9 h in the 2020s. Students reported low confidence in their ophthalmic knowledge and skills, with only 26.4% feeling confident in their knowledge and 34.5% in their skills.

#### 3.4.5 The future of ophthalmology education

Maling et al. (36) propose the need for global standards in ophthalmology training, citing significant variations in curricula, learning outcomes, and procedural requirements across different countries. They emphasize the importance of modern training tools such as AI, simulation, and virtual reality. The authors propose that establishing international standards could enhance the quality and consistency of ophthalmological education, ensuring better preparedness and skill proficiency among trainees.

## 4 Discussion

Vision has an enormous impact on quality of life. Blindness is ranked by the public to be the worst disease or ailment possible (37). Misdiagnosis and delayed diagnosis lead to demonstrated poor patient outcomes, including permanent vision loss, severe pain (38), and potentially loss of life. Thus, the ability to assess, interpret, and manage ophthalmic signs and ocular disease is essential to the training of safe medical practitioners. Understanding and surgical skills garnered in ophthalmic education is essential to the clinical competence.

The comprehensive analysis of various non-technical factors influencing ophthalmology education underscores the multifaceted challenges and opportunities within this field.

The COVID-19 pandemic has notably disrupted traditional educational practices, leading to heightened stress, anxiety, and depression among ophthalmic personnel and students. The shift to online learning and virtual platforms, while necessary, has yielded mixed results, highlighting the need for more effective and engaging educational methodologies. Economic crises, such as the Beirut explosion, have further compounded these challenges, exacerbating financial burdens and reducing clinical and surgical training opportunities. Significant heterogeneity in ophthalmology training exist in different regions. Major differences exist in surgical and medical competencies of doctors between Southeast Asia (SEA) and Western Pacific (WP). There were significant differences in the types of cataract surgeries performed between WP and SEA trainees. WP trainees performed more phacoemulsification surgeries (76, 0–500 vs. 19, 0–275, *P* = 0.004), extracapsular cataract extraction (34, 0–200 vs. 9, 0–50, *P* = 0.001), and intracapsular cataract extraction (1.5, 0–50 vs. 0.8, 0–15, *P* = 0.47) cases than their counterparts in SEA during their training. Gender dynamics play a crucial role in shaping career choices and educational experiences in ophthalmology, necessitating targeted mentorship programs to ensure equitable opportunities for all genders. Intrinsic motivation, driven by supportive educational environments that fulfill students’ psychological needs, is essential for effective learning and professional growth. Enhancing communication skills among residents can significantly improve patient cooperation and overall training effectiveness.

Persistent issues such as the lack of curricular and clinical rotation time, inadequate training resources, and the absence of standardized training curricula highlight the need for continuous adaptation and improvement in ophthalmology education. The decline in ophthalmology course lengths and the resultant low confidence among students in their ophthalmic knowledge and skills call for innovative teaching methods and increased clinical exposure. Looking forward, the establishment of global standards in ophthalmology training, coupled with the integration of modern training tools such as AI, simulation, and virtual reality, is imperative.

In recent years, a large number of novel techniques have emerged in ophthalmology. Among theses ophthalmic training, there were still non-technical factors that would influence ophthalmology education. Fatigue and loss of concentration over time may have affected residents’ performance (26). Insufficient financial support would pose a great barrier to establishing an advanced training curriculum (36). Lack of human resources in teaching could limit the utilization of the advanced training techniques to their full capacity (28). Therefore, multiple short sessions of training is suggested to the trainees, sufficient financial support and strong teaching resources are recommended to provide efficient ophthalmic training by the use of the novel techniques.

## 5 Conclusion

The non-technical factors that influenced ophthalmology education included various crisis, inadequate curricular time, training resources, lack of training standardization and shortage of financial support and teaching resources. The review summarizes the influences on ophthalmology education of various non-technical factors, thereby helping educators improve the training methods.

## Author contributions

YJ: Writing – original draft. HJ: Writing – original draft. ZY: Writing – review and editing. YL: Writing – review and editing. YC: Writing – review and editing.
